# Bruchpilot in Ribbon-Like Axonal Agglomerates, Behavioral Defects, and Early Death in SRPK79D Kinase Mutants of *Drosophila*

**DOI:** 10.1371/journal.pgen.1000700

**Published:** 2009-10-23

**Authors:** Vanessa Nieratschker, Alice Schubert, Mandy Jauch, Nicole Bock, Daniel Bucher, Sonja Dippacher, Georg Krohne, Esther Asan, Sigrid Buchner, Erich Buchner

**Affiliations:** 1 Department of Genetics and Neurobiology, Julius-Maximilians-University, Würzburg, Germany; 2 Institute of Anatomy and Cell Biology, Julius-Maximilians-University, Würzburg, Germany; 3 Department of Electron Microscopy, Julius-Maximilians-University, Würzburg, Germany; University of California San Francisco, United States of America

## Abstract

Defining the molecular structure and function of synapses is a central theme in brain research. In *Drosophila* the Bruchpilot (BRP) protein is associated with T-shaped ribbons (“T-bars”) at presynaptic active zones (AZs). BRP is required for intact AZ structure and normal evoked neurotransmitter release. By screening for mutations that affect the tissue distribution of Bruchpilot, we have identified a P-transposon insertion in gene *CG11489* (location 79D) which shows high homology to mammalian genes for SR protein kinases (SRPKs). SRPKs phosphorylate serine-arginine rich splicing factors (SR proteins). Since proteins expressed from *CG11489* cDNAs phosphorylate a peptide from a human SR protein *in vitro*, we name *CG11489* the *Drosophila Srpk79D* gene. We have characterized *Srpk79D* transcripts and generated a null mutant. Mutation of the *Srpk79D* gene causes conspicuous accumulations of BRP in larval and adult nerves. At the ultrastructural level, these correspond to extensive axonal agglomerates of electron-dense ribbons surrounded by clear vesicles. Basic synaptic structure and function at larval neuromuscular junctions appears normal, whereas life expectancy and locomotor behavior of adult mutants are significantly impaired. All phenotypes of the mutant can be largely or completely rescued by panneural expression of SRPK79D isoforms. Isoform-specific antibodies recognize panneurally overexpressed GFP-tagged SRPK79D-PC isoform co-localized with BRP at presynaptic active zones while the tagged -PB isoform is found in spots within neuronal perikarya. SRPK79D concentrations in wild type apparently are too low to be revealed by these antisera. We propose that the *Drosophila Srpk79D* gene characterized here may be expressed at low levels throughout the nervous system to prevent the assembly of BRP containing agglomerates in axons and maintain intact brain function. The discovery of an SR protein kinase required for normal BRP distribution calls for the identification of its substrate and the detailed analysis of SRPK function for the maintenance of nervous system integrity.

## Author Summary

Neurons communicate through release of neurotransmitters at specialized contacts called synapses. Modulation of synaptic transmission likely underlies all higher brain function including feature abstraction, learning and memory, and cognition. The complex molecular machinery that regulates neurotransmitter release has been conserved in evolution but is still incompletely understood. Using the genetic model organism *Drosophila*, we recently discovered a protein of the presynaptic ribbon (T-bar) that was called Bruchpilot (German for crash pilot) because flies with reduced amounts of this protein cannot fly. We now screened various *Drosophila* mutants for changes in tissue localization of Bruchpilot and discovered a gene that codes for an enzyme which is similar to mammalian kinases that phosphorylate splicing factors and may co-localize with Bruchpilot at the synapse. Larval nerves of mutants for this gene contain conspicuous accumulations of Bruchpilot that correspond to extensive electron-dense ribbon-like agglomerates surrounded by vesicles. While general axonal transport and basic synaptic transmission at larval nerve-muscle synapses are not affected, adult mutants show reduced life span and impaired flight and walking. The substrate for this kinase and its role in maintaining brain function must now be identified. Its discovery raises important questions about the function of homologous proteins in mammals including humans.

## Introduction

Molecular characterization of synaptic transmission has become a central theme of neuroscience research. A major contribution to the regulation of neurotransmitter release results from properties and interactions of proteins at the presynaptic active zone (AZ). In recent years several AZ components have been identified for vertebrates [1]–[5]. The large AZ specific proteins Piccolo and Bassoon together with RIM form a scaffold at the presynaptic AZ interacting with CAST/ERC, Munc13 and Liprin-α. Together these proteins form the cytomatrix at the active zone (CAZ) and are involved in regulation of the synaptic vesicle cycle and plasticity of synaptic transmission. Most presynaptic proteins have been conserved in *Drosophila* [6], with the notable exception of Piccolo and Bassoon. Sequence similarity between CAST/ERC and its insect homologue Bruchpilot (BRP) is largely confined to two N-terminal domains which in BRP are followed by extensive coiled-coil structures of about 1000 amino acids [7]. A present hypothesis proposes that these regions might convey cytoskeletal interactions that in vertebrates could be mediated by Piccolo and/or Bassoon [7]. Knock-down by RNAi or deletion of the *brp* gene leads to loss of the presynaptic dense projections (T-bars, [1]), reduction of calcium channel density, severe defects in synaptic transmission, and altered short-term plasticity [7],[8]. Stimulated emission depletion (STED) microscopy using the monoclonal antibody nc82 [9] which recognizes a C-terminal epitope of BRP revealed that BRP forms a donut-shaped ring centered at active zones [8]. No information is as yet available on the molecular mechanisms of AZ assembly in *Drosophila* while in vertebrates several active zone proteins are transported to the presynaptic terminal in the form of active zone precursor vesicles termed Piccolo transport vesicles (PTVs) [10]. Presumably, many more types of transport vesicles will be found, considering the complexity of vesicles observed near nascent active zones [11],[12].

Posttranslational modification of proteins of the CAZ has been suggested to be an important mechanism of synaptic modulation [13]. For example, mammalian serine/threonine kinase SAD-B is associated with synaptic vesicles and with CAZ, it phosphorylates RIM but not Munc13, and interference with SAD-B targeting inhibits synaptic transmission [14]. The *Drosophila* SAD homologue (encoded by *CG6114*) apparently has not yet been characterized. By screening for mutants with altered tissue distribution of BRP we now have identified a kinase presumably associated specifically with presynaptic active zones of *Drosophila*. It shows high homology to mammalian SR protein kinases. SR proteins are highly conserved phosphoproteins involved in the regulation of constitutive and alternative splicing [15]–[19]. SR proteins exhibit one or two N-terminal RNA-binding regions termed RRM (RNA recognition motif), as well as a serine/arginine (SR) rich C-terminal domain [20] which is required for protein-protein interactions affecting cellular localization [21] and regulation of splicing [22]–[24]. The genome of *Drosophila melanogaster* (*Dm*) encodes several SR proteins (SC35, SF2 (ASF), B52 (SRp55), RBP1, RBP1-like, X16, SRp54) [25]–[27]. Members of the highly conserved family of SR protein kinases (SRPKs) [28]–[35] have been shown to phosphorylate the RS (arginine/serine rich) domain of the SR family of splicing factors [29], [31], [36]–[39]. Phosphorylation modulates protein-protein or protein-RNA interactions and is therefore an important mechanism for the regulation of SR proteins. Three mammalian SRPKs have been described. Their characteristic feature is a bipartite highly conserved kinase domain interrupted by a unique spacer region involved in individual regulation [40]. SRPK1 and SRPK2 are widely expressed in mouse embryonic tissues while SRPK3 (Stk23) is a muscle specific protein kinase whose elimination leads to centronuclear myopathy [41]. Four *Srpk* genes have been detected in the *Drosophila* genome [25],[26],[42], *CG8174* at 52A1, *CG8565* at 13F3-4, *CG11489* (*CG9085*) at 79D4, and *Doa* (*CG1658*) at 98F6. Available information on the *Drosophila melanogaster (Dm)* gene *CG11489* includes its expression in the embryonic brain [43] and changes in transcript levels in various mutants [44],[45]. Here we further characterize the gene *CG11489* which we propose to name *Srpk79D* since sequence comparison identifies no clear autology to any of the three mammalian genes. We identify four alternatively initiated and spliced transcripts by RT–PCR, demonstrate that SRPK79D is able to phosphorylate a synthetic SR-substrate *in vitro*, and generate and characterize hypomorphic and null mutants for the *Srpk79D* gene. We show that mutation of this kinase gene leads to highly conspicuous accumulations of the active zone protein Bruchpilot (BRP) in discrete structures in larval and adult nerves. Functionally, elimination of SRPK79D causes locomotor defects and reduced longevity. Possible links between the lacking kinase activity, the structural abnormalities with altered BRP distribution, the behavioral defects, and the reduced life span are discussed.

## Results

In a screen of synaptic protein gene mutants to identify possible interaction partners of the active zone protein Bruchpilot we found that a small deletion affecting the *Csp* gene and the adjacent gene *CG11489* leads to conspicuous accumulations of Bruchpilot in discrete spots in larval nerves (cf. below). Other *Csp* alleles did not show these axonal BRP spots whereas the P-element *P{lacW}Csp^P2^* which actually inserts in *CG11489* did. We therefore decided to characterize this gene in more detail.

### The gene *CG11489* of *Drosophila melanogaster* codes for at least 4 different transcripts

**Figure 1:**
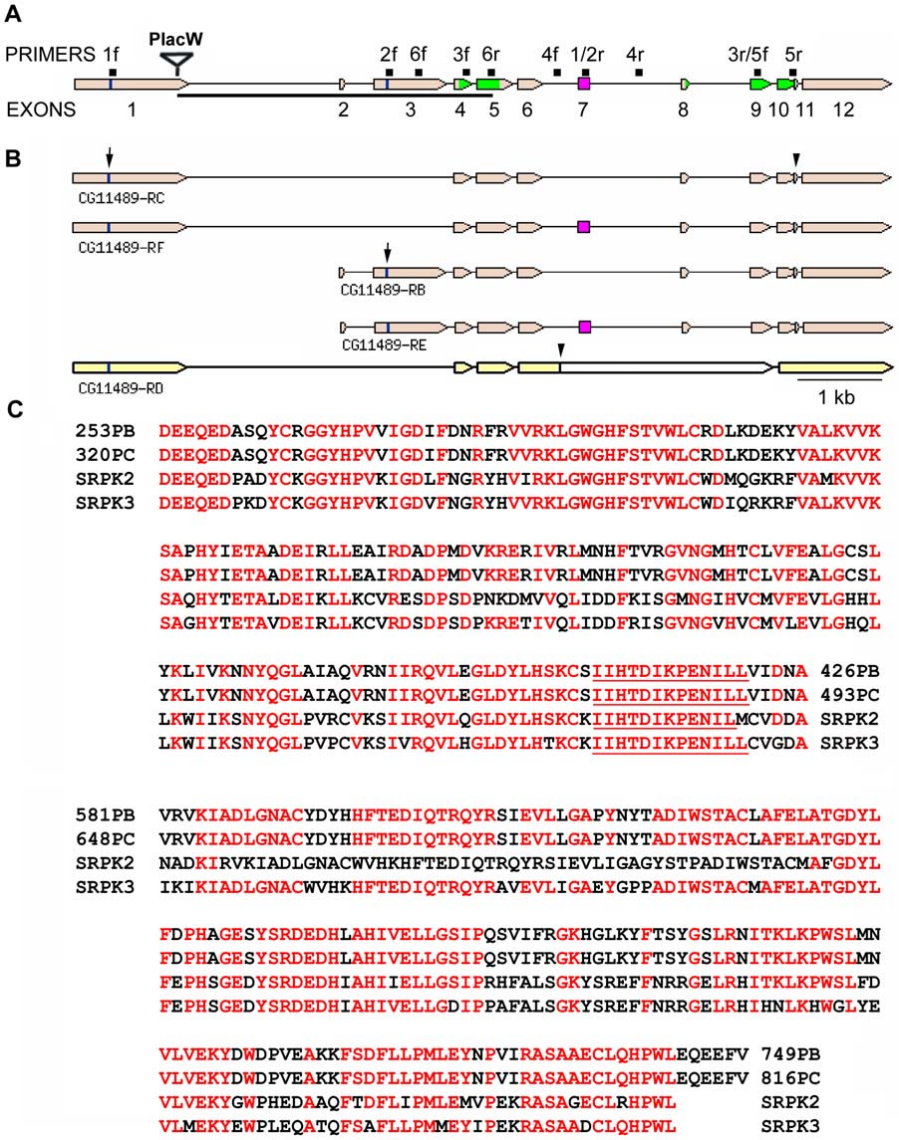
Structure of the *Srpk79D* gene, transcripts, and homology of kinase domain. (A) Exon-intron structure of the *Srpk79D* gene (*CG11489*) according to flybase modified due to the detection of the new exon 7 added (pink rectangle). The positions of the forward (f) and reverse (r) primers used for RT–PCR are indicated. The position of the P(lacW) insertion of the line *Srpk79D^P1^* is shown. It destroys the open reading frame of the RC/RF transcripts but leaves the RB/RE transcripts intact. This line is therefore considered a hypomorph. The P-jump-out mutant line *Srpk79D^VN^* suffered a deletion of 3861 bp indicated by the horizontal bar and contains a 23 bp remnant of the P-element. It is considered a null mutant. Sequences coding for the bipartite kinase domain are depicted in green. (B) The four transcripts inferred from RT–PCR with adult mRNA using the primer pairs indicated in (A). The two groups of transcripts RC/RF and RB/RE use different transcription and translation start sites (vertical bars marked by arrows) and are alternatively spliced. Stop codons are marked by arrowheads. The transcript RD of flybase is shown in a different color as it could not be verified using primer pair 4. The corresponding cDNA probably has been generated from an incompletely spliced RNA. (C) The two parts of the kinase domain of *Dm* SRPK79D (coding regions green in A) are separated by non-conserved spacers. The kinase domain is identical for all four protein isoforms (PC, PF, PB, PE) and shows high homology to human SR protein kinases SRPK3 and SRPK2 (and SRPK1, not shown). Identical amino acids are in red. The active center of the kinases is underlined.

According to the latest flybase update available from the Berkeley *Drosophila* Genome Project (BDGP) (http://flybase.org/, FB2009_01) the gene *CG11489* generates two transcripts RB and RD which use different transcription and translation start sites, share exons 4 and 5, but are differently spliced again downstream of intron 5 (Figure 1A and 1B). To reassess this information we sequenced wild-type Canton-S RT–PCR products obtained from adult mRNA by various primer combinations (Figure 1A). We were able to confirm the transcript RB and the transcript RC annotated in an earlier version of flybase. In addition we found a new exon (exon 7, 159 bp) that is alternatively spliced from both transcription starts, resulting in four RNA (R) transcripts (RB, RC, RE, RF) coding for four protein (P) isoforms of 749 (PB) and 802 (PE) and 816 (PC) and 869 (PF) amino acids (aa) as shown in Figure 1B. RT–PCR experiments designed to detect the RD transcript (coding for isoform PD of 695 aa) from adult poly-A^+^ mRNA were unsuccessful although control PCR from genomic DNA with the same primers generated reliable products.

### The gene *CG11489* codes for a serine/threonine protein kinase that shows high homologies to SR protein kinases

The *Drosophila* gene *CG11489* is predicted to code for serine/threonine protein kinase isoforms whose kinase domains consist of two parts, separated by a spacer of 155 or 208 amino acids (isoforms without or with the alternatively spliced exon 7, respectively) (http://www.expasy.org/prosite/). Homology searches (BLAST) reveal high homologies between these kinase domains and mammalian SR protein kinases (http://www.ncbi.nlm.nih.gov/). Since sequence comparisons fail to identify a clear autology relationship we propose to name *CG11489* the *Drosophila Srpk79D* gene. Figure 1C shows the homology for the kinase domain of *Dm* SRPK79D to human SRPK3 (65% aa identity) (MSSK1 [46]) and SRPK2 (54%) [47],[48]. Those parts of *Dm* SRPK79D that are not included in the kinase domain show no significant homologies to any other known proteins.

### *Dm* SRPK79D phosphorylates a synthetic SR protein peptide *in vitro*

**Figure 2:**
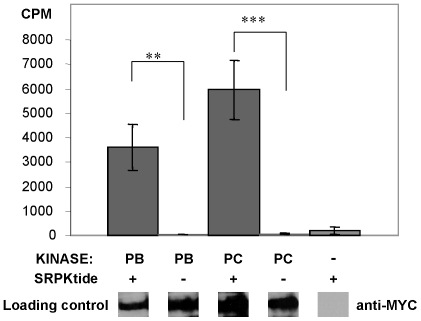
A peptide from a human SR protein (SRPK1tide) is a substrate for *Drosophila* SRPK79D *in vitro*. The two isoforms PC and PB of the SRPK79D differ in their N-terminal domain but are both able to phosphorylate the synthetic peptide SRPK1tide significantly. Measurements in all experimental groups were repeated five times independently. Statistical significance (Student's t-test with Bonferroni correction, **: p<0.01; ***: p<0.001) was tested against the corresponding groups without substrate, as indicated in the figure. Western blot analyses with anti-Myc antibody revealed that SRPK79D isoforms were loaded in all experimental groups, except in the negative control.

To investigate if SRPK79D is indeed a newly identified SR protein kinase in *Drosophila*, we performed *in vitro* phosphorylation assays with SRPK79D isoforms PC and PB using the synthetic peptide SRPK1tide (Upstate Chemicon) as a substrate. To isolate active *Dm* SRPK79D we transfected HEK293 cells with *myc*-tagged versions of *Srpk79D* cDNAs and purified the proteins by immunoprecipitation using an anti-Myc antibody. The substrate SRPK1tide corresponds to the amino acids 204–218 of the SR-rich region of the human SR protein ASF-1/SF-2 [15],[49]. Both tested isoforms of *Dm* SRPK79D are able to phosphorylate SRPK1tide significantly *in vitro* (Figure 2). Autophosphorylation could be excluded, because control preparations without substrate show no detectable phosphorylation. We therefore conclude that *Dm* SRPK79D is indeed an SR protein kinase.

### *Srpk79D* mutants

The *P{lacW}Csp^P2^* line was isolated in a screen to identify P-element insertions near the cysteine string protein gene (*Csp*) [50],[51]. By inverse PCR it was found that in this line the P(lacW) transposon inserted 81 bp upstream of the first exon-intron boundary of the *Srpk79D*-(RC/RF) transcripts and destroys the open reading frame of these mRNAs (Figure 1A). We therefore propose to rename this line as *Srpk79D^P1^*, short for *P{lacW}Srpk79D^P1^*. This line is considered a hypomorph because the two transcripts RB and RE remain intact and their expression is maintained at reduced levels as revealed by RT–PCR (data not shown). This P-element was remobilized and white-eyed jump-out lines were characterized by PCR and sequencing. The line *Srpk79D^VN^* suffered 3′ to the insertion site a deletion of 3861 bp (horizontal bar in Figure 1A) that includes the transcription and translation start of the *Srpk79D*-RB/RE transcripts and also eliminates the first 85 codons of the highly conserved SR kinase domain (Figure 1A). At the site of the deletion the line contains an insertion of 23 bp (remnant of the P-element). Thus this line is most likely a true null mutant. The line *Srpk79D^REV^* shows a precise excision of the P-element and is therefore considered a revertant.

### Mutations of the *Srpk79D* gene lead to accumulation of the active zone protein Bruchpilot in larval and adult nerves, but leave general axonal transport and basic synaptic structure and function intact

**Figure 3:**
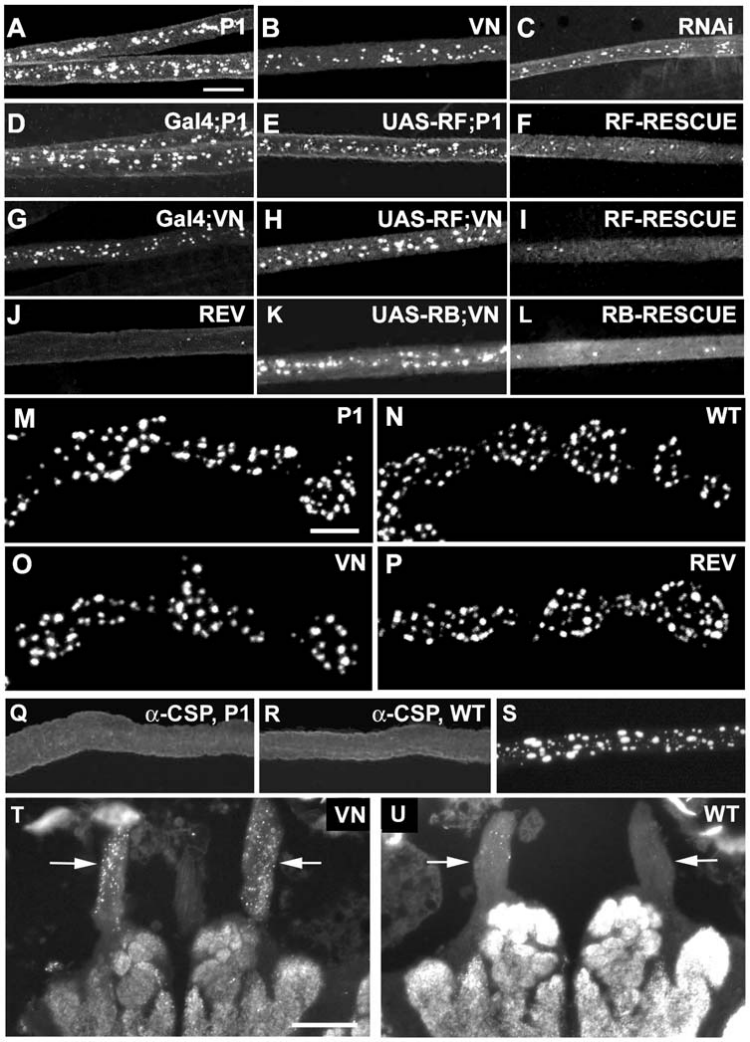
Mutation of the *Srpk79D* gene leads to the selective accumulation of the active zone protein BRP in axons of larval and adult nerves, but does not qualitatively change BRP distribution in synaptic boutons or neuropil. (A–I). Bruchpilot accumulations are observed in larval nerves of the hypomorph *Srpk79D^P1^* (A), the null mutant *Srpk79D^VN^* (B), an *elav*-driven RNAi line *elav-Gal4;;UAS-Srpk79D-RNAi* (C), the parental lines for the P1-rescue with the PF isoform, *w,elav-Gal4;;Srpk79D^P1^* (D) and *w;UAS-cDNA-RF;Srpk79D^P1^* (E), the parental lines for the null-rescue with the PF isoform, *w,elav-Gal4;;Srpk79D^VN^* (G) and *w;UAS-cDNA-RF;Srpk79D^VN^* (H), and the parental lines for the null-rescue with the PB isoform (G) and *w;UAS-cDNA-RB-eGFP;Srpk79D^VN^* (K). The revertant *Srpk79D^REV^* (J) and wild type (*w^1118^*, not shown) lack these structures. In the P1-rescue line *w,elav-Gal4;UAS-cDNA-RF;Srpk79D^P1^* (F) and the null-rescue lines *w,elav-Gal4;UAS-cDNA-RF;Srpk79D^VN^* (I) and *w,elav-Gal4;UAS-cDNA-RB-eGFP;Srpk79D^VN^* (L) the density of BRP accumulations is strongly reduced. (M–P). Typical chains of synaptic boutons type Ib stained with anti-BRP antibody nc82 on larval body wall muscles 6/7 of the hypomorphic mutant *Srpk79D^P1^* (M), wild type *w^1118^* (N), null mutant *Srpk79D^VN^* (O), and revertant *Srpk79D^REV^* (P). No difference in the distribution and qualitative expression levels of the presynaptic active zone protein BRP is seen between *Srpk79D* mutants and wild type. (Q–R). CSP does not accumulate in the larval nerves of the hypomorphic mutant *Srpk79D^P1^* (Q) or the null mutant (not shown). (R) *w^1118^* control. (S) Overexpression of BRP also leads to BRP accumulations in larval nerves. (T–U) BRP immunohistochemistry on frozen head sections of the null mutant *Srpk79D^VN^* (T) and wild type (U) reveals that massive spot-like BRP accumulations are observed in adult *Srpk79D^VN^* null-mutant antennal nerves (arrows in (T)). Larval preparations in (A–P) and (S) and the head sections in (T) and (U) were stained with monoclonal antibody (MAB) nc82 (anti-Bruchpilot). Larval nerves in (Q) and (R) were stained with MAB ab49 (anti-CSP). Scale bar in (A) for (A L), (Q–S): 10 µm. Scale bar in M for (M–P): 2 µm. Scale bar in (T) for (T–U): 50 µm.

Homozygous mutants, both of the hypomorphic *Srpk79D^P1^* allele and the *Srpk79D^VN^* null allele, show similar conspicuous accumulations of Bruchpilot in discrete spots in larval segmental and intersegmental nerves (Figure 3A and 3B). These BRP spots are also observed when *Srpk79D* mRNAs are knocked down by panneural expression of an RNAi construct directed against all four transcripts (Figure 3C). Whether these BRP spots represent aggregates of BRP or are accumulations of protein complexes containing BRP is not known at present. The axonal BRP accumulation phenotype is almost completely rescued by panneural overexpression of the SRPK79D-PF isoform in the hypomorphic mutant *Srpk79D^P1^* (Figure 3F) as well as in the null mutant *Srpk79D^VN^* (Figure 3I). The BRP accumulation phenotype is also rescued by transgenic expression of the SRPK79D-PB isoform (C-terminally linked to eGFP) (Figure 3L) or by the SRPK79D-PC isoform (C-terminally linked to eGFP) (data not shown) in the null mutant. The respective parental controls (Figure 3D, 3E, 3G, 3H, and 3K) display the mutant phenotype. Since in an independent study the SRPK97D-PB isoform is reported not to rescue the BRP accumulation phenotype of a different allele (*Srpk79D^ATC^*) of the kinase gene (Johnson et al., in press), we have repeated the RB rescue experiments and verified the UAS-RB cDNA transgene in the null mutant background by PCR using primer pair 6f/6r of Figure 1A which is specific for the RB/RE cDNAs and includes two introns thus allowing the distinction between genomic DNA and cDNA. Eight of eight larvae of the parental elav-Gal4 driver and 16 of 16 larvae of the parental UAS-RB cDNA line, both in *Srpk79D^VN^* null mutant background, showed the BRP accumulations as in Figure 3G and 3K, respectively, and 8 of 8 larvae of the F1 of this cross showed a clear, obvious rescue effect as in Figure 3L.

In vertebrates it was shown that proteins which build the cytomatrix at the active zone like Piccolo, Bassoon, RIM, Munc13-1 and N-Cadherin are transported by a special class of vesicles, the precursors of the active zone vesicles [10],[11],[52]. It thus seems possible that the axonal transport of either a special class of vesicles, or of the Bruchpilot protein itself is affected by the *Srpk79D* mutations. We therefore investigated whether the BRP accumulations in the nerve influence synaptic structure or the distribution of Bruchpilot at the active zones of *Srpk79D* mutant larvae. We analyzed the morphology of synaptic boutons and their active zones in *Srpk79D^P1^* or *Srpk79D^VN^* mutants and wild type on larval muscle 6/7 or 12/13 in segment A3. We could not detect a clear alteration in the gross morphology of the presynaptic boutons of the mutants (Figure 3M and 3O) compared to wild type (Figure 3N) or the revertant (Figure 3P) nor was there a significant difference in the number of boutons or of active zones in the null mutant in comparison to the wild-type controls (type Ib boutons of muscle 6/7 VN: 43.4±3.9, WT: 42.4±1.8, p>0.1, n = 8; active zones in type Ib boutons of muscle 12/13 VN: 222±27.9, WT: 275±31.8, p>0.1, n = 6). There was also no obvious difference in the distribution or the qualitative expression level of the Bruchpilot protein at the presynaptic active zones of larval motor neurons (Figure 3M–3P) (cf. Discussion).

Abnormal accumulations of proteins in the axon are often caused by general defects in axonal transport [53]–[58]. We therefore tested whether the accumulation of the Bruchpilot protein observed in the *Srpk79D* mutants might be due to a general impairment of the axonal transport machinery. We find that other synaptic proteins are uniformly distributed throughout the larval nerves of both wild-type and *Srpk79D^VN^* null mutant. This is illustrated for the synaptic vesicle protein cysteine string protein (CSP) [50] in the *Srpk79D^VN^* null mutant (Figure 3Q) and the *w^1118^* control (Figure 3R). In addition, other proteins of the presynaptic terminal like synaptotagmin or synapsin also do not accumulate abnormally in mutant nerves (data not shown), which allows us to conclude that axonal transport in general is not affected by the mutations of the *Srpk79D* gene. Finally, we tested whether a similar phenotype is also found in adult nerves. Figure 3T and 3U shows anti-BRP (nc82) staining of horizontal sections of adult null-mutant and wild-type brains, respectively. Neuropil regions of the antennal lobes and central protocerebrum are clearly labelled. In the mutant a large number of spot-like BRP accumulations are seen in both antennal nerves (arrows) whereas only a few spots are present in the antennal nerves of the wild type. This clear difference has been observed in four out of four pairs of preparations.

**Figure 4:**
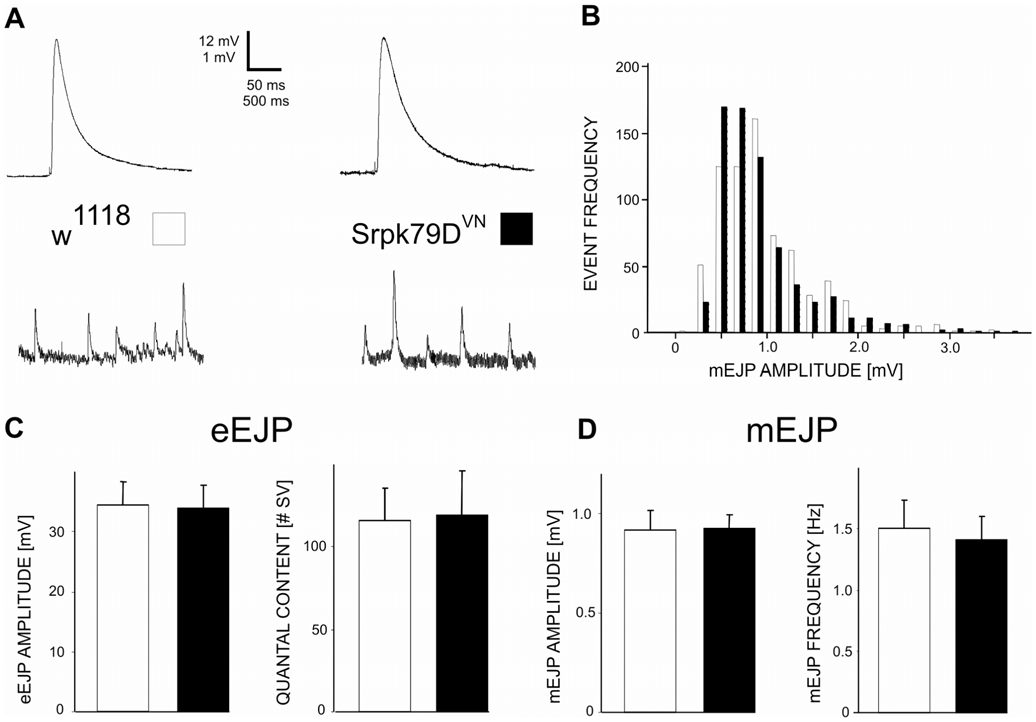
Basic synaptic transmission at the larval neuromuscular junction is normal in *Srpk79D^VN^* null mutants. Amplitude and time course of evoked EJP responses of *w^1118^* and *Srpk79D^VN^* null-mutant larvae are not significantly different ((A), upper diagrams and (C) left diagram) (*w^1118^* = 34.2±4.0 mV n = 9; *Srpk79D^VN^* = 33.8±3.7 mV; n = 7; p>0.05). Spontaneous single vesicle fusion events (mEJPs) have similar amplitude distributions (B) and similar mean amplitudes (*w^1118^* = 0.92±.1 mV and *Srpk79D^VN^* = 0.92±0.07 mV; n = 8; p>0.05) and frequencies (*w^1118^* = 1.5±0.2 Hz and *Srpk79D^VN^* = 1.4±0.2 Hz; n = 8; p>0.05) (D). Thus, quantal content is also not significantly different ((C), right diagram) (*w^1118^* = 116±19 vesicles and *Srpk79D^VN^* = 119±27 vesicles; n = 8; p>0.05). All data are means±SEM.

In line with the largely normal appearance of the mutant nervous system we find that basic synaptic transmission at the neuromuscular junction of *Srpk79D^VN^* null-mutant larvae, as reflected by amplitude and frequency of miniature excitatory junction potentials (mEJPs) and amplitude and quantal content of evoked EJPs (eEJPs) are not altered in comparison to wild-type controls (Figure 4). Thus we conclude that the accumulation of the Bruchpilot protein in larval nerves does not lead to major defects in the function of the presynaptic active zone and synaptic transmission at the larval neuromuscular junction.

### *Srpk79D* mutant larval nerves contain electron-dense agglomerates that contain Bruchpilot

**Figure 5:**
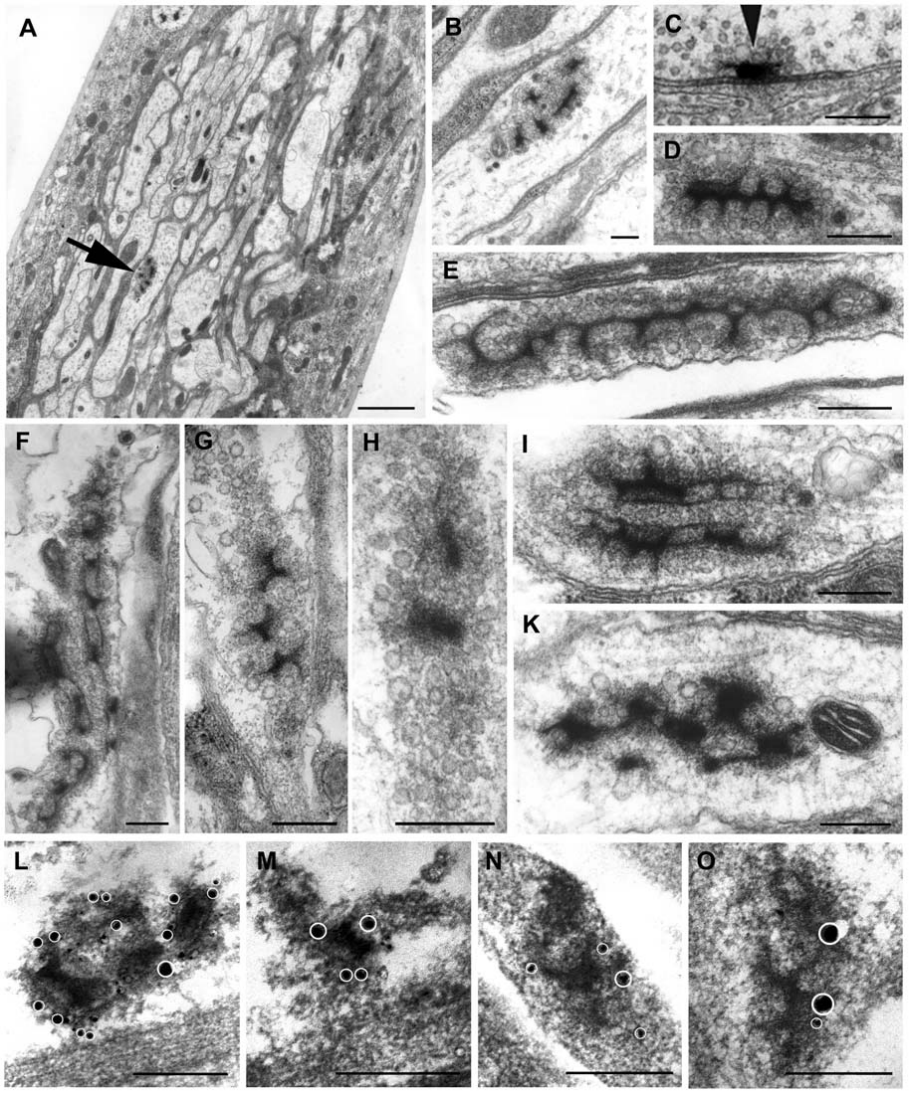
Mutation of the *Srpk79D* gene causes accumulation of BRP at extensive electron-dense ribbon structures in larval nerves. (A) Ultra-thin sections of a larval nerve of the *Srpk79D^VN^* null mutant. (B) Higher magnification of the conspicuous electron-dense structure in (A) (arrow). (C) For comparison a typical synaptic ribbon (T-bar, arrowhead) from a synaptic bouton of an *Srpk79D^+^* larval motor neuron terminal is shown. (D–K). Examples of large electron-dense structures observed in the mutant. (L–O) Silver-enhanced immuno-gold labelling using MAB nc82 (anti-BRP) as first antibody demonstrates that the electron-dense ribbon structures contain BRP. The silver precipitates (white circles) can be clearly recognized at enhanced brightness as shown for (L) in Figure S4. Scale bars in (A): 2 µm, in (B–O): 300 nm.

Since at the presynaptic terminal BRP is structurally and functionally associated with electron-dense synaptic ribbons (“T-bars”) [7],[8] we analyzed wild-type and *Srpk79D* null-mutant larval nerves emerging from the abdominal ganglia by standard electron microscopy in search for conspicuous electron-dense structures specific for the mutant. In each of three *Srpk79D* null-mutant larval preparations a nerve cross-section area between 6470 and 8717 µm^2^ was scrutinized, and per animal we found between 8 and 15 large electron-dense complexes of various shapes and varying diameters often surrounded by clear vesicles, as shown in the examples in Figure 5A, 5B, and 5D–5K. In three wild-type larvae an area between 6842 and 9750 µm^2^ each was analyzed. Here we found per animal between one and three small electron-dense structures of much lower complexity (not shown, Table 1). On average we found one electron-dense complex in 625.4 (±105.0; n = 3) µm^2^ in the mutant and one in 4904 (±1047; n = 3) µm^2^ in the wild type. Mean diameters of electron-dense complexes amounted to 449.04 nm (±43.5 nm; n = 37) in the mutant and 155.63 nm (±17.3 nm; n = 6) in the wild type. The electron-dense complexes in the mutant axons consist of complex ribbon-like structures resembling multiple T-bars and thus are considerably larger than a typical T-bar of a presynaptic larval neuromuscular bouton (shown in Figure 5C, arrowhead, for comparison). A rough estimate of the volume density of the complexes indicates that it is compatible with the density of accumulations of BRP observed by fluorescence microscopy with an antibody against BRP (Figure 3B). In order to verify that these electron-dense agglomerates correspond to the BRP containing spots seen in fluorescence microscopy we performed pre-embedding immuno-gold labelling of null-mutant axons. Figure 5L–5O shows examples of silver-enhanced gold particles (unequivocally identified at high image brightness and marked by white circles, cf. Figure S4) associated with electron-dense agglomerates similar to those shown in Figure 5A, 5B, and 5D–5K. We noticed that all gold particles observed on 14 agglomerates decorate the periphery of the electron-dense structures rather than their centers. We thus conclude that these agglomerates indeed contain BRP. The density and distribution of silver grains not associated with electron-dense agglomerates (background) are similar in wild-type and null-mutant larval nerves (not shown).

**Table 1:** Quantitative comparison of the number of electron-dense structures in wild-type and null-mutant larval nerves.

Animal	Area of the nerve sections analyzed (µm^2^)	Number of agglomerates
Wild type 1	9,242	2
Wild type 2	6,842	1
Wild type 3	9,750	3
Mutant 1	8,717	14
Mutant 2	6,674	15
Mutant 3	6,470	8

### Bruchpilot and overexpressed SRPK79D-PC and -PF but not -PB isoform co-localize at the presynaptic active zone

**Figure 6:**
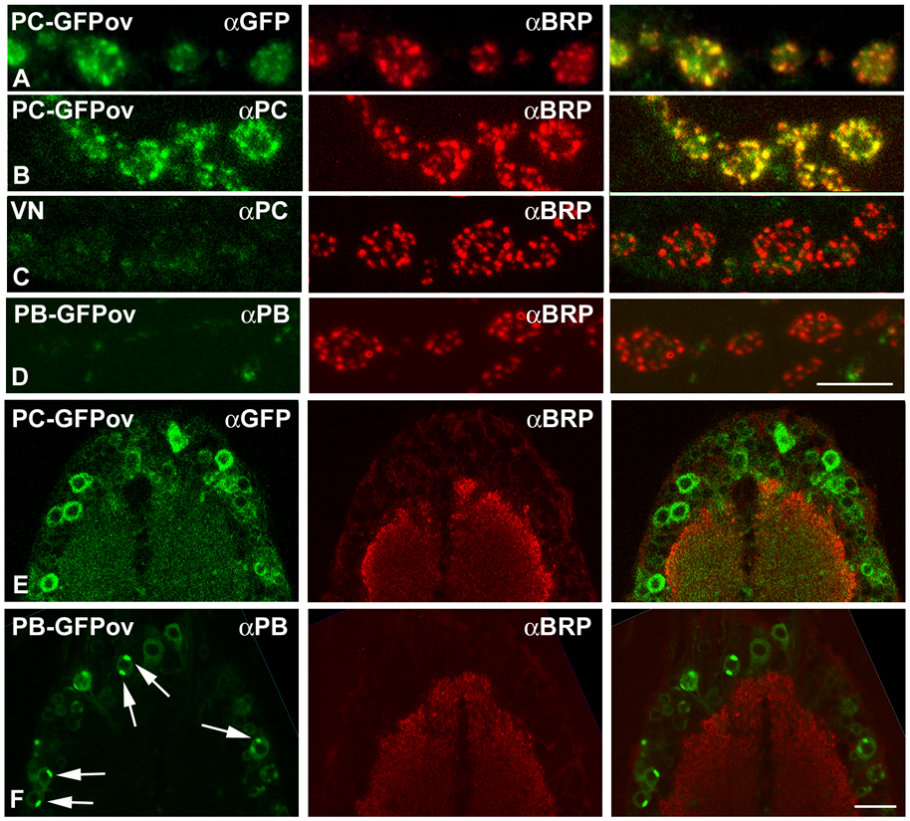
Overexpressed SRPK79D-PC and Bruchpilot co-localize at presynaptic active zones while overexpressed SRPK79D-PB accumulate in discrete perikaryal regions. The synaptic distribution of overexpressed SRPK79D-PC-GFP protein, stained with anti-GFP (A) or anti-PC (B) antisera (green, left) largely overlap with endogenous Bruchpilot stained with anti-BRP (nc82) (red, middle) at presynaptic active zones. Control stainings in the *Srpk79D^VN^* null mutant with anti-PC (C) demonstrate the specificity of the staining. No reliable synaptic staining is obtained with anti-PB antiserum (D). Both overexpressed isoforms are detected in the perikaryon, PC homogeneously ((E), left column), PB in discrete spots (arrows in (F), left column). BRP is not detectably localized in perikarya ((E), (F), middle column). Right column: overlay. Scale bar in (D) for (A–D): 5 µm. Scale bar in (F) for (E) and (F): 20 µm.

The finding that the accumulation of Bruchpilot protein in larval nerves of *Srpk79D* mutants is not due to a general impairment of the axonal transport machinery supports the hypothesis of a specific interaction of BRP and SRPK79D. However, a direct interaction of the two proteins is likely only if they can be shown to co-localize in the same cellular compartment. To investigate this, we generated affinity-purified antisera against the non-overlapping N-terminal parts of the SRPK79D-PB/PE (anti-PB) and –PC/PF (anti-PC) isoforms. The specificity of the antisera was tested by Western blots of adult heads of wild type, null mutant, and SRPK79D-PB-eGFP or –PC-eGFP overexpressing lines. In the overexpressing lines clear specific Western blot signals were obtained (Figure S1). From wild-type heads detectable amounts of the PC isoform could only be obtained after enrichment by immuno-precipitation (Figure S2). To characterize targeting of the kinase isoforms we panneurally overexpressed GFP-tagged SRPK79D-PB and -PC isoforms and performed double immuno-stainings on larval preparations with anti-BRP (nc82) and the anti-PB or anti-PC antisera or antibodies against GFP (anti-GFP). Figure 6A and 6B demonstrates that panneurally overexpressed SRPK79D-PC-eGFP and Bruchpilot co-localize at the presynaptic active zone. The specificity of the anti-PC staining is demonstrated by the parallel staining of the *Srpk79D^VN^* (VN) null mutant (Figure 6C). Therefore a direct interaction of the PC isoform and BRP at the presynaptic active zone seems possible. The PC isoform is in addition homogeneously distributed in the perikaryon (Figure 6E). The same distribution is observed for the overexpressed PF isoform (data not shown). Overexpressed SRPK79D-PB-eGFP, on the other hand, accumulates in discrete structures in the perikarya of larval neurons (Figure 6F) but is not targeted to synaptic terminals.

### Mutation of the *Srpk79D* gene impairs adult locomotion and causes short life span

**Figure 7:**
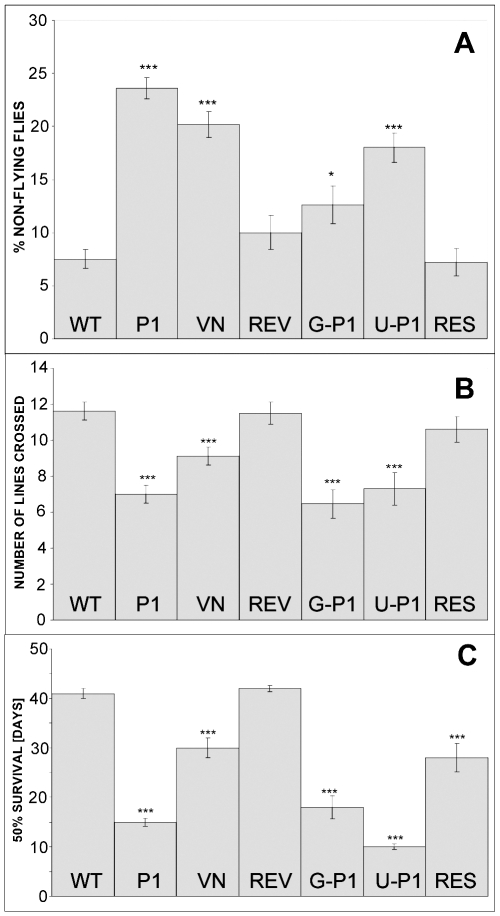
Mutation of *Srpk79D* gene results in behavioral deficits and reduced life span. (A) Compared to wild-type controls (WT) a significantly larger percentage of adult *Srpk79D^P1^* (P1) and *Srpk79D^VN^* (VN) mutants and parental flies for the rescue *w,elav-Gal4;;P1* (G-P1) and *w;UAS-RF;P1* (U-P1) drop to the bottom of a 500 ml glass cylinder when released at the top of the cylinder. Revertants (REV) and rescue flies *w,elav-Gal4;UAS-RF;P1* (RES) are not significantly different from wild-type. (B) Both mutants and the parental flies for the rescue also show lower spontaneous walking activity on a horizontal surface (wings clipped, number of lines of a 2×2 cm grid crossed during a 30 s period) whereas revertants and rescue flies are not impaired. (C) Longevity of both mutants and the parental flies for the rescue is significantly reduced. Rescue flies live significantly longer than their parents but not as long as wild type or revertants. Thus for longevity rescue is only partial. (Significance levels: ***: p<0.001; **: p<0.01; *: p<0.05, paired t-tests with Bonferroni correction of each column against WT).

With our antisera against SRPK79D-PB/PE and –PC/PF we could not detect any clear difference in immunohistochemical stainings between wild-type and null-mutant flies. (Overexpression led to similar observations as in larvae, i.e. synaptic neuropil staining with anti-PC serum and perikaryal spots with anti-PB serum, data not shown). However, by two behavioral tests and life span measurements we demonstrate that SRPK79D is required for intact nervous system function also in adults because the mutations in the *Srpk79D* gene lead to behavioral deficits in adult flies in addition to the BRP accumulation in nerves. Phenotypes of the *Srpk79D^P1^* (P1) and *Srpk79D^VN^* (VN) mutants include flight impairment (Figure 7A) and reduced ability or motivation to walk on a horizontal surface (Figure 7B) as well as reduced life span (Figure 7C). *Srpk79D^REV^* revertants (REV) behave like wild type. All three phenotypes, impaired flight, impaired walking, and reduced longevity were fully or partially rescued by transgenic panneural expression of the SRPK79D-PF isoform in the *Srpk79D^P1^* mutant background when compared to the parental controls *w,elav-Gal4;;P1* (G-P1) and *w;UAS-RF;P1* (U-P1) (Figure 7A–7C). The large survival difference between the P1 and the null mutant can most likely be assigned to genetic background effects because after extensive outcrossing of the *Srpk79D^P1^* line to wild type *w^1118^* this difference was no longer significant (data not shown). These genetic background effects could also explain why the rescue in *Srpk79D^P1^* mutant background did not extend the 50% survival time beyond the values found for the null mutant (Figure 7C “VN” and “RES”).

## Discussion

A considerable number of proteins located at the presynaptic active zone of both vertebrates and invertebrates have been characterized in recent years. However, little is known about their assembly into the characteristic active zone structure. The identification of the *Drosophila* active zone protein Bruchpilot [7], a large protein with significant homology to the vertebrate active zone protein CAST/ERC, has spawned interest in the study of active zone development, structure and function by using the highly accessible larval neuromuscular synapse of the genetic model organism *Drosophila*. Bruchpilot contains large coiled-coil regions similar to vertebrate proteins Piccolo and Bassoon that are lacking in *Drosophila*, and thus may combine functions of several vertebrate active zone proteins. Here we have described the identification and characterization of a kinase with high homology to vertebrate SR protein kinases (SRPKs) that is targeted to active zones and is required to prevent the accumulation of Bruchpilot in discrete spots within larval and adult nerves and the formation of large Bruchpilot containing electron-dense agglomerates in neuronal axons. Since the transcripts of the gene located in chromosomal subdivision 79D (and therefore termed *Srpk79D*) have been controversial in successive flybase releases we first analyzed cDNAs by RT–PCR.

### *Srpk79D* Transcripts

Using the information provided by the Berkeley *Drosophila* Genome Project (BDGP) [25] and flybase (http://flybase.org/, release January 2009) we confirmed by RT–PCR and sequencing the structure of the RB transcript and of the transcript RC that had been annotated in an earlier release. We extended this information by the detection of an alternatively spliced exon of 159 bp (exon 7 in Figure 1A). This newly identified exon is differentially included in both RB and RC transcripts generating the transcripts RE and RF, respectively. The 53 amino acids encoded by exon 7 are located in the non-conserved spacer region between the two highly conserved kinase sub-domains of the SRPK79D-PE and -PF isoforms. This spacer region was shown to play a role in the subcellular localization of yeast and human SRPKs [34]. We find that the overexpressed SRPK79D-PC and -PF (data not shown) but not the -PB isoform are targeted to synaptic active zones while the overexpressed -PB isoform accumulates in clearly defined perikaryal sub-regions (Figure 6). This demonstrates that in *Drosophila* the non-conserved N-terminal domain of SRPK79D isoforms that is generated by the use of alternative promotors contains important protein targeting information while the additional amino acids encoded by exon 7 for the spacer region do not alter the targeting observed here. Recently, an additional transcript (RD) has been annotated in flybase. This transcript shares the 5′ region with the RC and RF transcripts, however the entire genomic sequence between exons 6 and 9 is retained as a large exon (Figure 1B). Translation of this transcript would terminate in intron 6 and lead to a truncated protein of 695 amino acids which contains only the N-terminal half of the kinase domain and thus most likely would be non-functional. Our RT–PCR experiments did not detect the RD transcript in adult flies. The RD transcript is based on the existence of a cDNA clone (GH08190), which was generated in a high-throughput approach to produce cDNA clones for *Drosophila melanogaster* genes [59]. We propose that this cloned cDNA was reverse-transcribed from an incompletely spliced RNA. This interpretation is supported by the fact that the amino acids of the hypothetical PD isoform encoded by intron 6 show no homology to any known proteins and are not conserved among diptera.

### SR protein kinases

BLAST searches revealed that the kinase domain common to all four *Dm* SRPK79D isoforms shows high homology to the conserved family of SR protein kinases [27]–[34]. These kinases are known to be involved in the regulation of splicing via phosphorylation of SR proteins [28], [30], [35]–[38]. The kinase domain of all SR protein kinases consists of two highly conserved sub-domains, divided by a non-conserved spacer. Since homologies to all other kinases are considerably lower and a typical SR protein is readily phosphorylated by both *Dm* SRPK79D-PB and -PC isoforms we conclude that SRPK79D isoforms are members of the family of SR protein kinases. In *Drosophila melanogaster* there are three genes (*Srpk79D* (*CG11489*); *CG8174*; *CG8565*) with very high homology to the three human SR protein kinase coding genes (*hs Srpk1-3*) but little is known about the role of these genes in *Drosophila* so far. Recently the LAMMER kinase DOA (gene *CG33553*) which shows intermediate level of homology to SRPKs has been shown to phosphorylate the SR protein DX16 [26], increasing the number of putative *Srpk* genes in *Drosophila* to four. Highest amino acid identity of the kinase domain of *Dm* SRPK79D is observed with human SRPK3 (Figure 1C) which is specifically expressed in muscles [40]. However, the differences in homology to mammalian SRPKs are small and may not be significant. The various phenotypes of the *Drosophila Srpk79D* mutants and their rescue by panneural RC/RF-cDNA expression demonstrate that in *Drosophila* SRPK79D-PC/PF function is relevant in the nervous system. Antisera raised against the different N-terminal domains of PB/PE and PC/PF isoforms expressed in *E. coli* recognize the respective overexpressed proteins both in larvae and adults (Figure 6 and data not shown) but fail to detect the endogenous proteins, indicating that the expression level of the *Srpk79D* gene is very low. It is unlikely that posttranslational processing eliminates recognition of the endogenous kinase (PC/PF-isoform) by the anti-PC antiserum since a signal at the expected size can be detected after enrichment of the kinase by immunoprecipitation using the PC antiserum (Figure S2).

### SRPK79D function

In this report we show that mutation of the *Srpk79D* gene leads to accumulations of the active zone protein Bruchpilot in discrete spots in larval and adult nerves and demonstrate that these accumulations correspond in neuronal axons of larvae to large electron-dense structures surrounded by clear vesicles (Figure 5). The speculation that these electron-dense structures are molecularly related to T-bars is supported by their ribbon-like appearance, by the observation that the anti-BRP antibody nc82 binds to the periphery of the ribbons similar to what has been shown by STED microscopy for synaptic T-bars [8], and by the fact that they apparently are able to bind clear vesicles. Photoreceptor terminals of Bassoon knock-out mice contain synaptic ribbons detached from active zones. These “floating” ribbons are associated with vesicles, presumably synaptic vesicles [60]. Also, in axons of young rat hippocampal cultures aggregates of dense-core and clear vesicles are observed which label positively for synaptic vesicle markers like synaptobrevin, SV2, synaptotagmin and synapsin-I [61]. We excluded that the clear vesicles associated with the axonal agglomerates described here are mature synaptic vesicles because they are not labelled by various antibodies against synaptic vesicle proteins, like cysteine string protein (CSP), synapsin, synaptobrevin, and synaptotagmin. No difference in the staining of larval nerves between wild type and *Srpk79D* mutants is observed with these antibodies (Figure 3Q and 3R, and data not shown). These experiments also exclude a general impairment of the axonal transport machinery as cause for the BRP accumulation phenotype because synaptotagmin and CSP have been shown to accumulate in the axons of mutants known to affect axonal transport [53]–[57]. Also, light microscopical morphology of the larval neuromuscular junction and the qualitative distribution of BRP as reflected by the number of presynaptic boutons and the number of BRP-positive active zones is not altered in our *Srpk79D* mutants compared to wild type. We have not attempted to quantify the amount of BRP at the active zones. In a report published simultaneously a different mutant allele *Srpk79D^ATC^* of the *Srpk79D* gene is characterized which contains a P-element insertion in intron 8 of the gene and thus disrupts all four transcripts. This mutation causes very similar accumulations of BRP in larval nerves and the authors report a 30% reduction of BRP immuno-fluorescence at the larval neuromuscular active zones in homozygous *Srpk79D^ATC^* mutants [62]. This observation is interpreted as an impairment of BRP transport to the presynaptic active zone of larval neuromuscular junctions due to a premature assembly of T-bar-like agglomerates in peripheral nerves [62].

Our immunohistochemical studies revealed that transgenically overexpressed GFP-tagged SRPK79D-PC and -PF (but not –PB) isoforms co-localize with Bruchpilot at the presynaptic active zone (Figure 6A–6C and data not shown). This observation indicates either that the N-terminus of SRPK79D-PC and -PF isoforms contains targeting signals for active zone localization or that these kinase isoforms can bind to active zone proteins during transport. Thus, a direct interaction of SRPK79D-PC/PF and BRP at the active zone seems possible although co-immuno-precipitation experiments for the two proteins were unsuccessful (data not shown). The obvious question of whether there are SR proteins at active zones and whether RNA splicing can occur at presynaptic active zones has now to be investigated. There is increasing evidence that presynaptic mRNA translation may contribute to synaptic plasticity [63],[64]. However, larval olfactory conditioning [65] of *Srpk79D^VN^* null mutants was not significantly impaired (Figure S5). Since overexpressed GFP-tagged SRPK79D-PB is not found at active zones but nonetheless rescues the BRP accumulation phenotype in larval nerves of *Srpk79D^VN^* null mutants our data do not support the hypothesis that mRNA splicing at active zones might be required to prevent the axonal BRP accumulations.

We have not observed a clear functional difference for the different SRPK79D isoforms. The striking axonal BRP accumulation phenotype is seen both in the *Srpk79D^P1^* mutant (lacking isoforms PC/PF and showing reduced expression of isoforms PB/PE) and in the *Srpk79D^VN^* null mutant (lacking all isoforms). Since it can be rescued in both mutants by all three available rescue cDNA constructs, RB, RC and RF (Figure 3F, 3I, 3L, and data not shown), we conclude that *the expression level* of the kinase is important, not which N-terminus it contains nor apparently whether it is localized at the active zones. Whether this is also true for the behavioral and survival phenotype must remain open since the corresponding rescue experiments were performed only with *Srpk79D^P1^* mutants overexpressing the RF cDNA (Figure 7). The reasons why the BRP accumulation phenotype of our deletion mutant *Srpk79D^VN^* is rescued by our RB cDNA construct but not the very similar phenotype of the *Srpk79D^ATC^* allele of Johnson et al. (in press) by their RB cDNA construct has now to be investigated.

Interestingly, mutation of the serine/threonine kinase Unc-51 that recently has been shown to regulate the localization of Bruchpilot to sites opposing the glutamate receptor fields in the postsynaptic membrane also causes BRP accumulations in larval nerves similar to the ones described here [66]. These authors interpret the BRP accumulations as axonal transport defects. However, a general axon transport defect can be excluded for the *Srpk79D* mutants. Yet another condition leading to BRP accumulations in larval nerves similar to the ones described here is the overexpression of BRP itself (Figure 3S). We excluded that mutation of the *Srpk79D* gene influences the expression level of the *brp* gene by semi-quantitative RT–PCR (data not shown) and by immuno-blotting (Figure S3). We also found no evidence for changes in splicing of *brp* transcripts in the *Srpk79D* mutant by RT–PCR (data not shown) or by Western blotting (Figure S3). Thus we speculate that an unknown factor co-transported with BRP along neuronal axons may have to be present at a correct stoichiometric ratio with BRP to prevent the axonal assembly into the large electron-dense structures seen in the electron microscope (Figure 5). This ratio can be disturbed by BRP overexpression or by reduced expression of the unknown factor. Alternative splicing to regulate transcription factor activities and hence gene expression (e.g. for the unknown factor) is a general mechanism known from early *Drosophila* development or sexual differentiation and from cell cycle regulation [67]. By this hypothesis a link between the available information about SRPK79D function and the larval BRP accumulation phenotype could be proposed. Attempts to prevent the formation of BRP accumulations by simultaneous overexpression of both BRP and SRPK79D-PC failed (Figure S6). However, the correct wild-type stoichiometric ratio of the unknown factor and BRP is perhaps difficult to restore.

The adult *Srpk79D* mutant flies show general behavioral impairments like locomotor defects and reduced life time (Figure 7). The effect of the *Srpk79D* mutation on the distribution of BRP in adults is similar to the larval phenotype in that BRP accumulates in various nerves (Figure 3T and data not shown). The expressivity of this phenotype is however rather variable such that further experiments are required to clarify the cause of this variability. Thus at present we have no evidence that the altered BRP distribution is indeed responsible for the behavioral phenotypes. Altered expression of the unknown factor mentioned above due to defective splicing could of course explain these phenotypes. Since no clear electrophysiological defects are observed in larval nerve-muscle preparations of the *Srpk79D* null mutants (Figure 4) we cannot offer a more specific hypothesis. On the other hand, subtle synaptic defects which may well play a role in central brain network function cannot be excluded because they may go unnoticed at the robust neuromuscular junction with its huge complement of reserve pool vesicles [68]. Also, since larval nerves contain both sensory and motor axons, we cannot be certain that the BRP accumulations are found in motor axons. The typical large BRP accumulations are not seen in the motor axons just before they branch to form the synaptic boutons. However, the fact that the behavioral defects of the *Srpk79D^P1^* mutant are reverted after precise excision of the P-element insertion and can be fully or partially rescued by panneural expression of the SRPK79D-PF isoform in the mutant (Figure 7) clearly link the phenotype to the *Srpk79D* gene and rule out genetic background effects. The rescue experiments also demonstrate that the eGFP moiety at the C-terminus does not impair the SRPK79D function required for prevention of the BRP accumulations. The observation that the rescue of the axonal BRP accumulation (Figure 3F and 3I) and of the reduced life span (Figure 7C) is incomplete, may be due to the fact that the three other isoforms are missing in the rescue animals or to the incorrect amount and/or distribution of the transgenically expressed protein.

### Conclusions

The present results demonstrate an important role of the kinase SRPK79D for the proper distribution of the active zone protein Bruchpilot. In larval and adult nerves the kinase is required for preventing the formation of conspicuous BRP-containing electron-dense ribbon-like agglomerates observed by electron microscopy in the *Srpk79D^VN^* mutant but not in wild-type controls. It is tempting to speculate that these ribbons may be molecularly related to T-bars beyond the association with BRP and that the kinase prevents the premature assembly of T-bars in peripheral axons [62]. Whether BRP is also involved in generating the behavioral and survival defects observed when SRPK79D-PC/PF isoforms or all SRPK79D isoforms are missing is not known. Since BRP does not contain any serine-arginine rich domains it seems unlikely that BRP is a substrate for these kinases. Our *in vitro* phosphorylation data suggest that in *Drosophila* SRPK79D isoforms modify SR proteins and thus may be involved in splicing regulation. It will now be necessary to identify the endogenous substrates of the SRPK79D kinase and study the mechanisms by which the formation of the extensive BRP-containing electron-dense agglomerates in wild-type axons is prevented. The characterization of an SR protein kinase that appears to be localized at presynaptic active zones and has dramatic effects on the distribution of an active zone protein is likely to modify current views on vertebrate SRPK function and may initiate new approaches to the study of active zone assembly and function.

## Materials and Methods

### Fly stocks

*P{lacW}Csp^P2^* line was generated in this lab [50],[51], *w^1118^*, *w,elav-Gal4*, *w;actin-Gal4/CyO*, *Δ2–3^Ki, p^* jump starter and balancer lines were obtained from the Bloomington Stock Center, *Srpk79D*-RNAi line (ID 8451) was obtained from the Vienna *Drosophila* RNAi Collection. Flies were maintained at 25°C or 18°C under a 14/10 h light/dark cycle at 60–70% relative humidity.

### RNA preparation, cDNA synthesis, PCR, and sequencing

Total RNA was isolated using the QIAGEN RNeasy Mini Kit (Qiagen; Hilden; Germany). Before reverse transcription of the messenger RNA into cDNA a 1 hour DNase digestion (RNase-free DNase, Roche; Mannheim; Germany) was performed at 37°C. The reverse transcription was performed with SuperScriptII Reverse Transcriptase (Invitrogen; CA; USA) following the instruction manual. The oligo-dT-primer used was purchased from MBI Fermentas (oligo(dT_18_), MBI Fermentas; St.Leon-Rot; Germany) and the dNTPs were from Metabion (Metabion international AG; Planegg-Martinsried; Germany). The cDNA of the *Srpk79D* gene was amplified using different sets of specific primers (from Eurofins MWG GmbH; Ebersberg; Germany). PCR was performed with Phusion High-Fidelity DNA Polymerase (Finnzymes; Espoo; Finland). The following primer pairs were used for the transcript analysis (cf. Figure 1A):

1f: 5′ACGAGAATTCGATGGCCGGCCTCATC3′

1r: 5′GTACGGTGTTGGGCTTG3′

2f: 5′CGGCGAATTCGATGGATGACTTTGGCT3′

2r: 5′GTACGGTGTTGGGCTTG3′

3f: 5′AAAAGCTTACCGGTTTCGAG3′

3r: 5′TCGAAGGCCAAACAGGC3′

4f:5′GTTGTGGTGTGCATGGAAAG3′

4r: 5′CAATCATATATGTAGGTGTGGCCA3′

5f: 5′GCCTGTTTGGCCTTCGA3′

5r: 5′AAAGCGGCCGCGACGAACTCCT3′

For verification of the parental RB rescue line UAS-RB-cDNA-GFP in *Srpk79D^VN^* null mutant background the primer pair

6f: 5′ GCGACTTCAACTTCGTCTCC 3′

6r: 5′ GCGGATTATGTTACGCACCT 3′

was used which gives a 1240 bp product for the wild-type gene and a 1086 bp product for the cDNA transgene. PCR products were purified with QIAGEN PCR Purification Kit (Qiagen; Hilden; Germany) and the DNA-Fragments were sequenced by Eurofins MWG GmbH (Eurofins; MWG; Ebersdorf; Germany).

### Cell culture, immunoprecipitation, and western blot analysis

Human embryonic kidney (HEK) 293 cells were grown at 37°C in the presence of 7% CO_2_ and cultured in Dulbecco's modified Eagle's medium (DMEM, Biowest; Nuaillé; France) containing 10% fetal calf serum. Transfection with *myc*-tagged *Srpk79D* cDNA constructs was performed with PolyFect reagent (Qiagen; Hilden; Germany) following the instruction manual. Cells were harvested in PBS, 48 h after transfection, and lysed in 400 µl lysis buffer containing 25 mM Tris pH 7.5, 150 mM NaCl, 2 mM EDTA, 2 mM EGTA, 10% Glycerol, 0.1% Nonidet NP-40 supplemented with protease inhibitors (complete mini EDTA, Roche; Mannheim; Germany) at 4°C for 40 min. 300 µg of protein lysate were incubated for 10 min with 0.8 µg of mouse monoclonal anti-Myc antibody (9E10, Santa Cruz; Heidelberg; Germany) in a total amount of 500 µl lysis buffer. Protein complexes were precipitated over night at 4°C with 50 µl protein-A-sepharose (Invitrogen; Carlsbad; CA; USA). The beads were washed three times with lysis buffer (without protease inhibitors). The proteins were eluted from the sepharose by incubation with Laemmli buffer and separated by SDS-PAGE, transferred to nitrocellulose membranes and probed with mouse monoclonal anti-Myc antibody (9E10, Santa Cruz; Heidelberg; Germany. The proteins were visualized using HRP-coupled secondary antibodies and the ECL detection reagent (Amersham Biosciences; Braunschweig; Germany). For immunoprecipitation from adult heads using antisera 1000 flies (volume∼3 ml) were frozen in liquid nitrogen, heads were isolated by sieving and homogenized in 800 µl of lysis buffer supplemented with protease inhibitor. The supernatant of a 30 min centrifugation at 16,000 g was incubated with antiserum (1∶1000 f.c.) for 10 min before 100 µl of protein-G agarose beads were added for over night incubation. For immunoprecipitation using hybridoma supernatant 50 adult flies were frozen in liquid nitrogen, heads were isolated and homogenized in 500 µl of lysis buffer supplemented with protease inhibitor. The supernatant of a 30 min centrifugation at 16,000 g was incubated with the hybridoma supernatant (1∶100 nc82) for 10 min before 100 µl of protein-G agarose beads were added for over night incubation. In both cases the beads were washed three times with lysis buffer and after centrifugation 40 µl of Laemmli buffer was added to the pellet and heated to 96°C for 5 min. The sample was analyzed by Western blotting.

### *In vitro* kinase assays

Cells were transfected, harvested and lysed as described above. Immunoprecipitated Myc-SRPK79D isoforms were washed five times in lysis buffer (without protease inhibitor), once in 500 µM NaCl and twice in kinase buffer containing 8 mM MOPS-NaOH, pH 7.0, and 0.2 mM EDTA. For the kinase reaction, 5 µl kinase buffer, 2.5 µl SRPK1tide (3 mM, Upstate Chemicon; Lake Placid; NY; USA) as a substrate, 5 µl H_2_O, and 10 µl ATP-Mix (500 µM ATP, Roche; Mannheim, Germany), 0.75 µCi γ^32^ATP (10 µCi/µl, 3000 Ci/mmol; GE Healthcare Life Sciences; Braunschweig; Germany) dissolved in 25 mM Mg(CH_3_COO)_2_ were added to the SRPK79D beads, and the mixture was incubated for 30 min at 30°C. The reaction was stopped by spotting the sample onto p81 phosphocellulose paper (diameter 2.5 cm, Whatman International LTD; Maidstone; UK). The papers were washed three times for 15 minutes in 175 mM H_3_PO_4_ to remove unbound ATP, before transferring them to scintillation vials containing 3 ml H_2_O. The amount of radioactive ^32^P incorporated into the substrate was determined in a scintillation counter (LKB wallac 1214 Rackbeta, Liquid Scintillation counter) by measuring the Čerenkov radiation. Each experimental condition was tested five times. Samples without kinase and samples without substrate served as negative controls. Significance was calculated performing the Student's t-test with Bonferroni correction.

### Mutagenesis of the *Srpk79D* gene

In the line *Srpk79D^P1^* (*P{lacW}Csp^P2^*) a P{lacW}-element is inserted in the first exon 81 bp upstream of the 3′ exon-intron boundary of the *Srpk79D*-RC/RF transcripts, as was verified by PCR and sequencing. Flies of this line were crossed to “jump-starter” flies (*Δ2–3^Ki, p^*). The offspring was crossed to *w;;TM3/TM6* balancer flies. The F2 generation was screened for individuals with white eyes and either *TM3* or *TM6*. 600 lines were established as balanced stocks from these individuals. Homozygous flies were subjected to PCR to characterize deficiencies produced by the P-element remobilization. The following primers were used: Forward: CGGCCGGCATATGTAGTAGT; reverse: GCGGATTATGTTACGCACCT. The breakpoints of the deletion in the *Srpk79D* gene were identified by sequencing with the same primers. The SRPK79D null mutant *Srpk79D^VN^* suffered a deletion of 3861 bp.

### Cloning of transgenic *Srpk79D* cDNA constructs

To restore the wild-type phenotype in *Srpk79D* mutants the complete *Srpk79D*-RF cDNA was cloned into the pP[UAST]-vector [69] and the construct was transformed into the germ line of *white* (*w* ^−^) *Drosophila* by standard techniques [70]. Transgene insertions on the 2^nd^ chromosome were recombined with either the *Srpk79D^P1^* or the *Srpk79D* ^VN^ mutation. Rescue experiments were performed with the offspring of these flies crossed to the *Gal4* driver line *elav-Gal4* (on X chromosome), in the corresponding *Srpk79D* mutant background. Thus these flies express the SRPK79D-PF isoform in the entire nervous system.

To facilitate the subcellular localization of SRPK79D isoforms fusion constructs of the entire *Srpk79D*-RB, -RC, and RF cDNAs in frame with the complete sequence of the eGFP cDNA were cloned into the pP[UAST]-vector [69]. The eGFP sequence was amplified from the vector pMes-EGFP [71] and fused to the N-terminus for the –PF and to the C-terminus for the PB and PC isoforms. The constructs were transformed into the germ line of *Drosophila* and expression of the different SRPK79D-eGFP fusion proteins was driven with *elav-Gal4* or *actin-Gal4* in the nervous system.

### Generation of antisera

The cDNAs coding for the respective first exons were amplified by PCR using the primers:

for RB/RE: EcoRI sense 5′GGGAGAATTCATGGATGACTTTGGCTC3′

XhoI anti 5′AAAACTCGAGCTCTTCCTTGACCGG3′

for RC/RF: EcoRI sense 5′AAAGAATTCATGGCCGGCCTCATC3′

XhoI-anti: 5′AAAACTCGAGAGACTGACGAATGGGCCG3′

The amplified sequences were cloned into the TOPO-vector (TOPO TA cloning Kit, Invitrogen; Carlsbad; CA; USA). The fragments were excised using EcoRI and XhoI and cloned in frame with a His-tag into the pET-21a+ expression vector (Novagen; Bad Soden; Germany).

The protein was expressed in *E. coli* BL21 and purified by using nickel-chelate affinity chromatography following the protocol (Qiagen; Hilden; Germany). Polyclonal guinea pig antibodies were generated [72]. Whole blood was collected, and the serum was separated for antibody purification.

### Affinity antibody purification

For the antibody purification the protein was expressed in *E. coli* BL21 and precipitated with chloroform/methanol. Following the protocol for affinity column HiTrap NHS-activated HP 1 m (GE Healthcare; Buckinghamshire; England) the column was equilibrated with buffer A and B before loading. Next the serum diluted in PBS was applied to the column. After washing steps the antibodies were eluted with Glycin (pH 2.5) and neutralized with Tris/HCl (pH = 8.9). The antibodies were concentrated using CentriPlus 50 Centifugal Filter Units (Millipore; Schwalbach; Germany). For stabilization 4 mg/ml BSA (Sigma-Aldrich; Schnelldorf; Germany) and 0.1% NaN_3_ was added.

### Immunohistochemistry

Wandering third instar larvae were dissected in ice-cold calcium-free *Drosophila* saline containing 128 mM NaCl, 35 mM Sucrose, 2 mM KCl, 4 mM MgCl_2_, 3 mM HEPES, pH 7.2, 1 mM EDTA, pH 7.0. The larvae were pinned down, cut open dorsally along the midline and gut and fat body were removed. The preparations were fixed in 4% paraformaldehyde pH 7.4 for 1.5 hours on ice. Fixative was prepared as follows: 2 g paraformaldehyde was dissolved in 25 ml H_2_O at 60°C for five minutes. Then 100 µl of 1N NaOH was added. After cooling down to room temperature 25 ml of 2× PEM buffer (200 mM PIPES, 4 mM EGTA, 1 mM MgSO_4_, pH 7.0) were added and the pH was checked. After fixation the preparations were washed 3 times for 15 minutes in PBST (PBS containing 0.1% Triton-X 100) at room temperature. Non-specific binding was blocked by incubating with a blocking solution (2% BSA (Sigma-Aldrich; Schnelldorf; Germany), 5% normal serum (Vector Laboratories; Burlingame; CA; USA) in PBST) for 1 hour at room temperature. Incubation with the primary antibody was performed over night at 4°C. Primary antibodies were nc82 (anti-Bruchpilot [7]), ab49 (anti-CSP [50]) and anti-GFP (rabbit) (Dianova; Hamburg; Germany). Nc82 and ab49 were diluted 1∶100, anti-GFP 1∶1000 in blocking solution. Before incubation with the secondary antibody, unbound primary antibody was removed by washing with PBST (3 times 45 minutes) at room temperature. Incubation with the secondary antibody (goat anti-mouse IgG Cy3) or goat anti-rabbit IgG Alexa488 (Molecular Probes; Karlsruhe; Germany) was performed at room temperature in the dark for 1 h. Secondary antibodies were diluted 1∶1000 in PBST. Then unbound secondary antibody was removed by washing in PBST for 4 times 1 h in the dark. Finally, preparations were embedded in Vectashield (Vector Laboratories; Burlingame; CA; USA). Scans were performed with a confocal laser scanning microscope and raw data were processed with Image J.

The preparation of adult frozen head sections has been described [9]. Briefly, air sacs and proboscis were removed in ice-cold 4% paraformaldehyde to allow quick access of the fixative to the brain. Flies were fixed at 4°C for 3 hours. Next they were incubated over night in 25% sucrose in *Drosophila* ringer serving as washing solution and freeze protectant. Fly heads were embedded in 16% carboxymethylcellulose (low viscosity, Sigma-Aldrich; Schnelldorf; Germany) and frozen in liquid nitrogen. 10 µm thick cryosections were collected on pre-chilled slides (SuperFrost Plus, Menzel-Glaser GmBH) and air-dried at RT for 20 minutes. Slides were blocked with normal serum for 2 h at RT and incubated with the first antibody (nc82, mouse monoclonal supernatant, dilution 1∶100) over night at 4°C. After washing twice with PBST for 10 minutes, the secondary antibody (goat anti-mouse, Alexa 488, dilution 1∶1000, Invitrogen) was applied for 1 hour at room temperature. After washing the sections twice 10 minutes in PBST they were mounted in Vectashield.

### Electrophysiology

Recordings were made from muscle fiber 6 in abdominal segments A3–A5 of third-instar wandering larvae after the peripheral nerves had been cut from the ventral ganglion. Dissections were performed in calcium-free hemolymph-like Ringer's HL3 solution [73]: 70 mM NaCl, 5 mM KCl, 20 mM MgCl_2_, 10 mM NaHCO_3_, 5 mM trehalose, 115 mM sucrose, and 5 mM HEPES, with a pH of 7.2. Recordings were performed in HL3, to which calcium was added to a final concentration of 1 mM. Intracellular muscle potential was recorded using a 1600 Neuroprobe amplifier (A-M Systems Inc.; Carlsborg; Washington; USA). Recording electrodes (borosilicate glass 1 mm OD/0.58 mm ID) with resistance of 10–20 MΩ were filled with 3 M KCl. Responses were recorded from muscle fibers with resting potentials between −58 and −76 mV. Data was low-pass filtered at 10 kHz, digitized, and acquired with a DAP card (Microstar Laboratories; Bellevue; Washington; USA) and recorded with DASYlab (National Instruments Ireland Resources Ltd.; Moenchengladbach; Germany). Mean evoked EJP amplitude was calculated from 60 consecutive EJPs elicited at 1 Hz. Evoked EJP amplitude and decay kinetics were analyzed using FORTRAN and verified with DASYlab. Miniature EJPs were recorded in 1 minute bins and analyzed with DASYlab. Quantal content was calculated by the ratio of eEJP/mEJP amplitudes after correcting eEJPs for nonlinear summation [74].

### Electron microscopy

Wandering third instar larvae were dissected in *Drosophila* saline as described for immunohistochemistry. The preparations were fixed with buffered 2.5% glutaraldehyde for one hour at 4°C and rinsed five times with 50 mM cacodylate buffer. Post-fixation was accomplished with 4% buffered osmium tetroxide for 90 minutes on ice. Preparations were washed five times with ddH_2_O on ice and stained *en bloc* with 0.5% aqueous uranyl acetate over night at 4°C. They were washed five times with ddH_2_O on ice and were dehydrated with graded series of ethanol. (50%, 70%, 90%, 96% and two times 100% ethanol at 4°C for 30 minutes each, fresh 100% ethanol and three times propylene oxide at room temperature for 30 minutes each). The preparations were then incubated in a 1+1 mixture of Epon (Serva Electrophoresis GmbH; Heidelberg; Germany) and propylene oxide over night and twice in pure Epon for two hours at room temperature. Finally, the larvae were embedded in Epon and polymerized at 60°C for 48 hours. Longitudinal ultrathin (80 nm) sections of the larval bundles of axons were cut using a diamond knife. The grids were post-stained with 2% uranyl acetate for 20 minutes and with Reynold's lead citrate for seven minutes.

For quantitative analysis sections spaced more than 1.5 µm apart were selected to avoid counting the same electron-dense structure multiple times. The nerve sections were analyzed with a Leo 912 AB transmission electron microscope (Zeiss SMT; Oberkochen; Germany) at 630× magnification and the cross-section area was measured with the polygon tool of iTEM software (Soft Imaging System; Muenster; Germany). Nerves were screened for conspicuous electron-dense structures at 40000× magnification. These were digitally photographed and their position in the nerve was marked at 16× magnification. Identification and counting of electron-dense structures were done blinded. The diameter of the agglomerates was measured with iTEM as the largest distance in the electron dense field. Mean values and standard error of the mean (SEM) were calculated.

### Pre-embedding immuno-gold labelling

For ultrastructural localization of Bruchpilot, wandering wild-type and null-mutant (*Srpk79D^VN^*) larvae were prepared in ice-cold calcium-free saline (130 mM NaCl, 36 mM Sucrose, 5 mM KCl, 1.9 mM MgCl_2_, 5.5 mM HEPES, 0.5 mM EDTA), fixed in 2% paraformaldehyde with 0.06% glutaraldehyde in 1× PEM (0.1 M PIPES, 2 mM EGTA, 1 mM MgSO_4_×7H_2_O) for 90 min on ice, washed twice for 15 min each in 1× PEM, blocked for 1 h in 2% BSA/3% normal horse serum (NHS) in PBS containing 0.2% Triton-X 100 and incubated overnight at 4°C with the primary monoclonal antibody nc82 diluted 1∶100 in PBST. Larval filets were then washed four times for 1 h in PBST, incubated for 1 h with the secondary antibody Alexa Fluor488 FluoroNanogold-anti-mouse Fab' (Nanoprobes; Yaphank; NY; USA) diluted 1∶20 in PBST, washed for 30 min in PBST and then washed overnight at 4°C in PBST. After washing twice for 30 min in PBST, the preparations were post-fixed for 30 min in 2% glutaraldehyde in PBS and washed four times for 10 min in distilled H_2_O. After the silver enhancement performed for 1 h using the AURION R-GENT SE-EM Kit, the preparations were washed four times for 10 min in distilled H_2_O, fixed for 30 min in 2% OsO_4_ in 50 mM cacodylate buffer (pH 7.2) and washed overnight at 4°C in distilled H_2_O. After washing twice for 30 min in distilled H_2_O and dehydration in ascending ethanol series (30 min each in 50%, 70%, 90%, 96% on ice; twice 100% at room temperature), the tissue was incubated twice for 30 min in propylene oxide and then in a 1∶1 mixture of propylene oxide and Epon overnight, followed by two 2-h incubation periods in pure Epon. The tissue was then transferred to gelatine capsules with pure Epon and polymerization was allowed to proceed for 3 days at 60°C. Ultrathin (70 nm) sections were cut and transferred to copper grids which were then contrasted with 2% uranyl acetate for 20 min, washed and incubated in Reynold's lead citrate for 10 min and subjected to a final wash.

### Behavioral assays

The walking and the flying assays were performed as described [7]. Briefly, for the walking assay individual flies with clipped wings were allowed to walk on a horizontal surface marked with a 2×2 cm grid. The number of lines crossed within 30 s was counted and recorded 3 times for each fly. At least 25 flies of each genotype were tested. In the flight assay groups of 100 flies were tossed through a funnel into a 500 ml cylinder (of 50 mm diameter) whose walls had been coated with paraffin oil. Poor fliers drop to the bottom of the cylinder and were counted. 10 groups were tested for each genotype. For life span analyses a total of at least 500 flies were tested. 50 to 100 newly hatched male flies were transferred to fresh food vials every three to four days and the dead flies were counted. Males were chosen because survival of isolated males is less variable than survival of females or mixed populations. Wild-type controls were *w^1118^* since it represents the genetic background of all experimental lines. Values are given as mean and SEM. Statistical significance of the difference to wild type was calculated using the Student's t-test with Bonferroni correction for 6 comparisons in each set of experiments. Larval olfactory conditioning was assayed as described [65]. Briefly, a group of 30 to 35 larvae was exposed for 5 min to vapor from undiluted 1-octanol (O^+^) while crawling on 1% agarose containing 2 M fructose, transferred to pure 1% agarose and exposed for 5 min to vapor from n-amyl acetate (A) (diluted 1∶50 in paraffin oil). This procedure was repeated 3 times, after which they were transferred to pure 1% agarose in the presence of antagonistic gradients of both odors. After 3 min the number of larvae closer to each odor source was counted and the preference index PI_O+_ = (# near O−# near A)/total # was calculated. Next a new group of larvae was treated equivalently, only now A instead of O was presented in the presence of fructose and PI_A+_ was calculated. The total procedure was repeated 10 times and the learning index LI = (PI_O+_−PI_A+_)/2 was calculated. Prior to the experiment the *Srpk79D^VN^* mutant had been backcrossed to wild type CS for 6 generations to minimize genetic background effects. The experiments were done blind with respect to the genotype.

## Supporting Information

Figure S1 Antisera against SRPK79D-PC and -PB recognize the respective overexpressed isoforms. Western blots of head homogenates of wild type (WT), *Srpk79D^VN^* null mutant (VN), and of flies overexpressing under the control of *elav-Gal4* the SRPK79D isoforms -PC-eGFP (PCov, left blot) and -PB-eGFP (PBov, right blot). Antisera against the PC-isoform (left blot) or the PB isoform (right blot) recognize their antigen only when it is overexpressed. MAB ab49 (anti-CSP) was used to visualize the loading control signal at 32–36 kDa.
(0.57 MB TIF)

Figure S2 Immunoprecipitation using antiserum against SRPK79D-PC. The anti-PC serum precipitates its antigen from wild-type (WT) head homogenates. The null mutant *Srpk79D^VN^* (VN) serves as a negative control. HC and LC mark signals from heavy and light chains of the precipitating antibodies.
(0.17 MB TIF)

Figure S3 BRP expression levels and isoforms are not altered in *Srpk79D* mutants. No increase of BRP expression (left blot) or change in BRP isoforms detected by MAB nc82 is observed in head homogenates of *Srpk79D^P1^* mutants (P1) or null mutants (VN) compared to wild type (WT). The blots were developed with anti-BRP (MAB nc82), the left blot in addition with anti-SAP47 (MAB nc46) as a loading control. Each IP lane contains 6 head equivalents. HC and LC mark signals from heavy and light chains of the precipitating antibodies.
(0.60 MB TIF)

Figure S4 Identification of silver-enhanced immuno-gold particles. Here Figure 5L is shown enlarged and at enhanced brightness to illustrate the discrimination of silver precipitates from ribbon-like agglomerates.
(2.93 MB TIF)

Figure S5 Larval olfactory conditioning is not significantly disturbed in *Srpk79D^VN^* null mutants. Larvae alternately exposed to 1-octanol in the presence and to n-amyl acetate in the absence of fructose (or vice versa) prefer the previously rewarded odor as indicated by a positive learning index. Learning indices are plotted as median with 25%–75% boxes and 10%–90% whiskers. No significant difference (p>0.15, n = 10, Mann-Whitney U-test) is found between wild type Canton-S (WT) and *Srpk79D^VN^* null mutants (VN).
(0.63 MB TIF)

Figure S6 Simultaneous overexpression of SRPK79D isoforms with BRP does not rescue the larval axonal BRP accumulation phenotype of flies overexpressing BRP. Larval progeny of crosses w,elav-Gal4;; either with w;UAS-Srpk79D-RB-eGFP;UAS-BRP (A) or with w;UAS-Srpk79D-RC-eGFP;UAS-BRP (B) both show the typical spot-like BRP accumulations indicating that increased levels of either kinase isoform cannot cure the axonal BRP accumulation effect observed whenever BRP is overexpressed (as shown in Figure 3S).
(0.20 MB TIF)
