# Supplementary figures and images for: Bruchpilot in Ribbon-Like Axonal Agglomerates, Behavioral Defects, and Early Death in SRPK79D Kinase Mutants of Drosophila
Source: PLoS Genet. 2009 Oct 23;5(10):e1000700. doi: 10.1371/journal.pgen.1000700 (PMC2759580; doi:10.1371/journal.pgen.1000700)

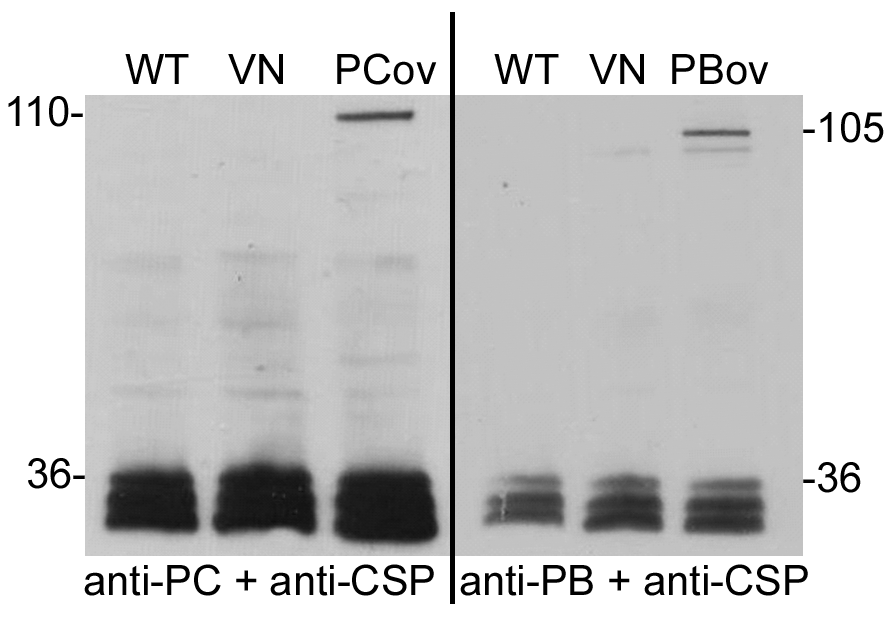

Supplement: Figure S1 — Antisera against SRPK79D-PC and -PB recognize the respective overexpressed isoforms. Western blots of head homogenates of wild type (WT), Srpk79DVN null mutant (VN), and of flies overexpressing under the control of elav-Gal4 the SRPK79D isoforms -PC-eGFP (PCov, left blot) and -PB-eGFP (PBov, right blot). Antisera against the PC-isoform (left blot) or the PB isoform (right blot) recognize their antigen only when it is overexpressed. MAB ab49 (anti-CSP) was used to visualize the loading control signal at 32–36 kDa. (0.57 MB TIF) [file pgen.1000700.s001.tif]

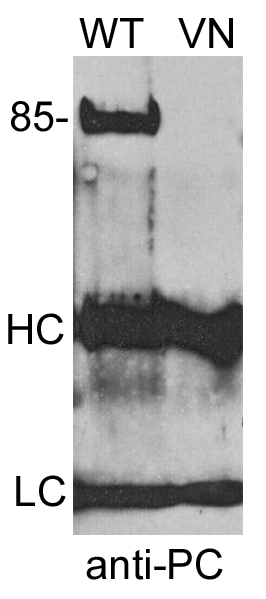

Supplement: Figure S2 — Immunoprecipitation using antiserum against SRPK79D-PC. The anti-PC serum precipitates its antigen from wild-type (WT) head homogenates. The null mutant Srpk79DVN (VN) serves as a negative control. HC and LC mark signals from heavy and light chains of the precipitating antibodies. (0.17 MB TIF) [file pgen.1000700.s002.tif]

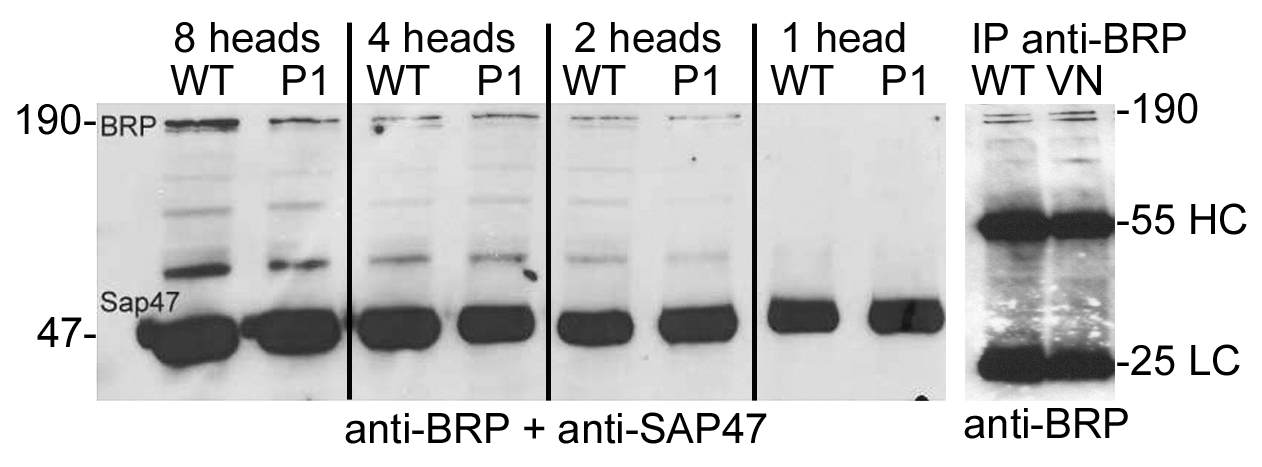

Supplement: Figure S3 — BRP expression levels and isoforms are not altered in Srpk79D mutants. No increase of BRP expression (left blot) or change in BRP isoforms detected by MAB nc82 is observed in head homogenates of Srpk79DP1 mutants (P1) or null mutants (VN) compared to wild type (WT). The blots were developed with anti-BRP (MAB nc82), the left blot in addition with anti-SAP47 (MAB nc46) as a loading control. Each IP lane contains 6 head equivalents. HC and LC mark signals from heavy and light chains of the precipitating antibodies. (0.60 MB TIF) [file pgen.1000700.s003.tif]

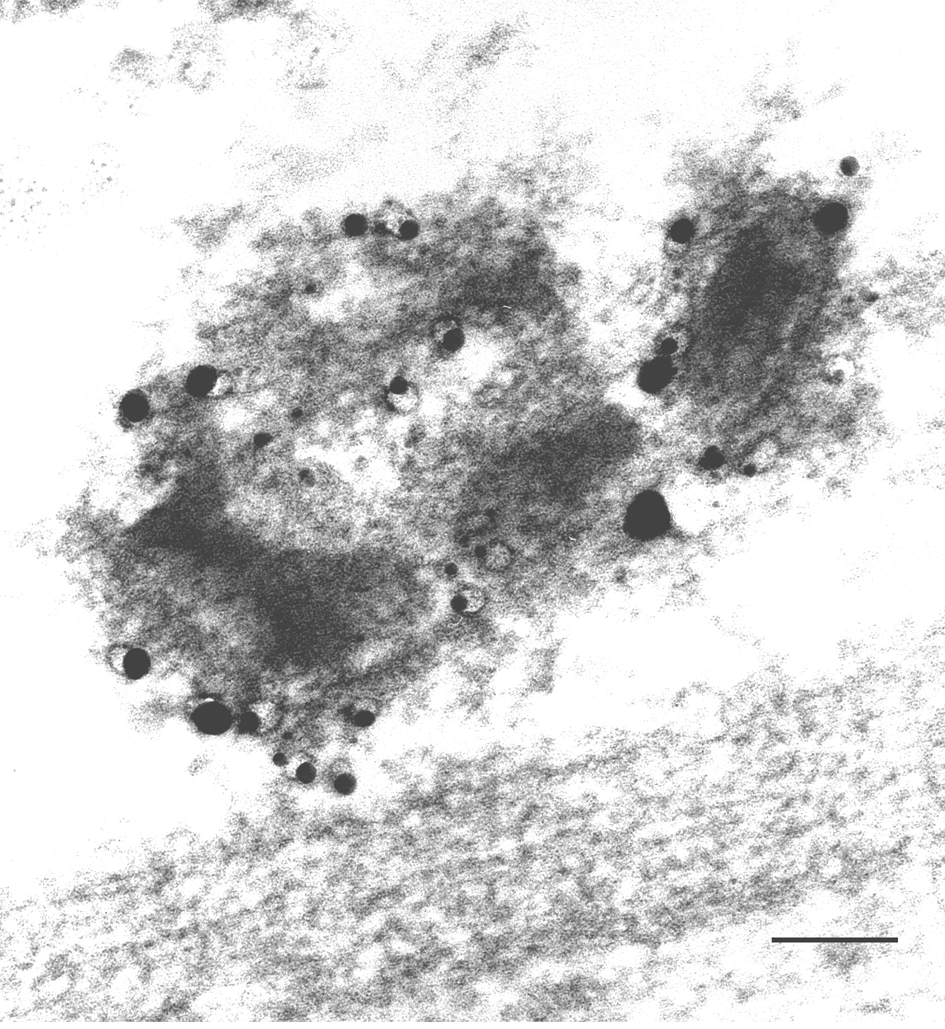

Supplement: Figure S4 — Identification of silver-enhanced immuno-gold particles. Here Figure 5L is shown enlarged and at enhanced brightness to illustrate the discrimination of silver precipitates from ribbon-like agglomerates. (2.93 MB TIF) [file pgen.1000700.s004.tif]

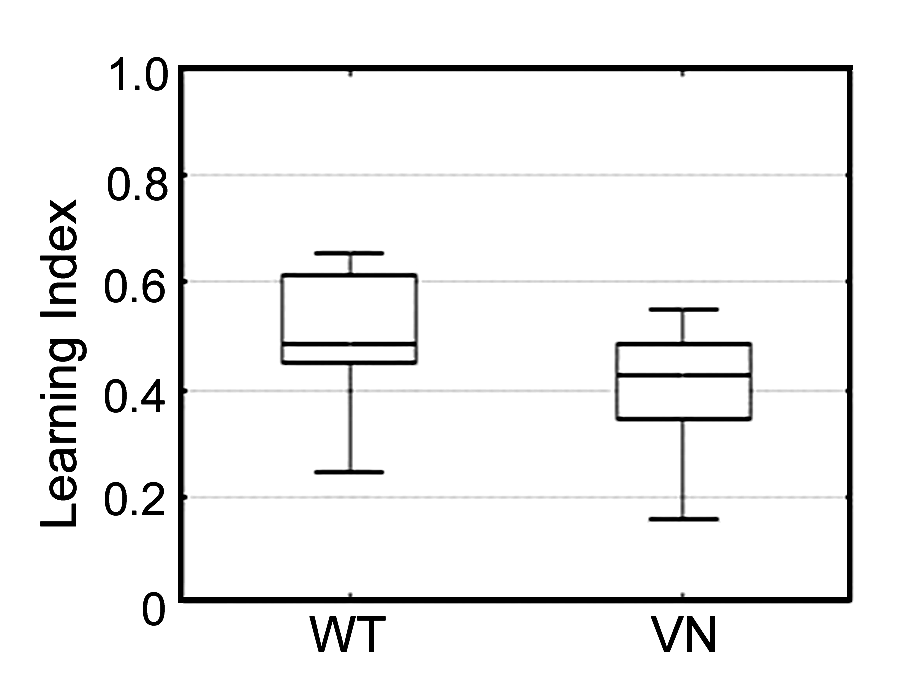

Supplement: Figure S5 — Larval olfactory conditioning is not significantly disturbed in Srpk79DVN null mutants. Larvae alternately exposed to 1-octanol in the presence and to n-amyl acetate in the absence of fructose (or vice versa) prefer the previously rewarded odor as indicated by a positive learning index. Learning indices are plotted as median with 25%–75% boxes and 10%–90% whiskers. No significant difference (p>0.15, n = 10, Mann-Whitney U-test) is found between wild type Canton-S (WT) and Srpk79DVN null mutants (VN). (0.63 MB TIF) [file pgen.1000700.s005.tif]

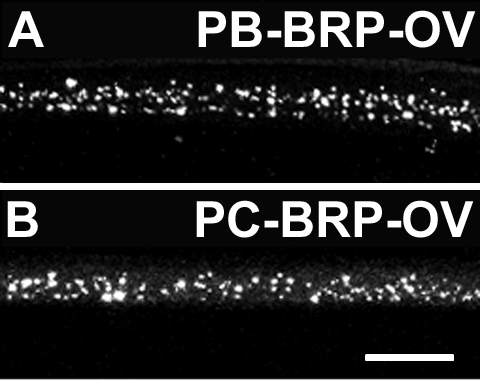

Supplement: Figure S6 — Simultaneous overexpression of SRPK79D isoforms with BRP does not rescue the larval axonal BRP accumulation phenotype of flies overexpressing BRP. Larval progeny of crosses w,elav-Gal4;; either with w;UAS-Srpk79D-RB-eGFP;UAS-BRP (A) or with w;UAS-Srpk79D-RC-eGFP;UAS-BRP (B) both show the typical spot-like BRP accumulations indicating that increased levels of either kinase isoform cannot cure the axonal BRP accumulation effect observed whenever BRP is overexpressed (as shown in Figure 3S). (0.20 MB TIF) [file pgen.1000700.s006.tif]
